# Efficient gene editing in *Corynebacterium glutamicum* using the CRISPR/Cas9 system

**DOI:** 10.1186/s12934-017-0814-6

**Published:** 2017-11-14

**Authors:** Feng Peng, Xinyue Wang, Yang Sun, Guibin Dong, Yankun Yang, Xiuxia Liu, Zhonghu Bai

**Affiliations:** 1 National Engineering Laboratory for Cereal Fermentation Technology, Jiangnan University, Wuxi, 214122 China; 2 The Key Laboratory of Industrial Biotechnology, Ministry of Education, School of Biotechnology, Jiangnan University, Wuxi, 214122 China; 3 The Key Laboratory of Carbohydrate Chemistry and Biotechnology, Ministry of Education, School of Biotechnology, Jiangnan University, Wuxi, 214122 China

**Keywords:** CRISPR/Cas9, *Corynebacterium glutamicum*, Genome editing, Protein expression

## Abstract

**Background:**

*Corynebacterium glutamicum* (*C. glutamicum*) has traditionally been used as a microbial cell factory for the industrial production of many amino acids and other industrially important commodities. *C. glutamicum* has recently been established as a host for recombinant protein expression; however, some intrinsic disadvantages could be improved by genetic modification. Gene editing techniques, such as deletion, insertion, or replacement, are important tools for modifying chromosomes.

**Results:**

In this research, we report a CRISPR/Cas9 system in *C. glutamicum* for rapid and efficient genome editing, including gene deletion and insertion. The system consists of two plasmids: one containing a target-specific guide RNA and a homologous sequence to a target gene, the other expressing Cas9 protein. With high efficiency (up to 100%), this system was used to disrupt the *porB*, *mepA*, *clpX* and Ncgl0911 genes, which affect the ability to express proteins. The *porB*- and *mepA*-deletion strains had enhanced expression of green fluorescent protein, compared with the wild-type stain. This system can also be used to engineer point mutations and gene insertions.

**Conclusions:**

In this study, we adapted the CRISPR/Cas9 system from *S. pyogens* to gene deletion, point mutations and insertion in *C. glutamicum*. Compared with published genome modification methods, methods based on the CRISPR/Cas9 system can rapidly and efficiently achieve genome editing. Our research provides a powerful tool for facilitating the study of gene function, metabolic pathways, and enhanced productivity in *C. glutamicum.*

**Electronic supplementary material:**

The online version of this article (10.1186/s12934-017-0814-6) contains supplementary material, which is available to authorized users.

## Background

*Corynebacterium glutamicum*, a gram-positive bacterium with a high G+C content, has been used for the industrial production of various amino acids for more than 50 years. Moreover, it has recently demonstrated strong potential for use as a protein expression system [1, 2] because of its excellent culture characteristics and also because it is non-pathogenic, does not produce endotoxins, and is generally recognized as safe [3, 4]. Furthermore, *C. glutamicum* produces minimal protease activity in the culture supernatant and has the ability to secrete properly folded proteins, which can improve subsequent purification efficiency. However, compared with *Escherichia coli* (*E. coli*), *C. glutamicum* has some intrinsic disadvantages, e.g. much lower transformation efficiency and lower levels of protein expression [4, 5]; comprehensive genetic and physiological investigations are needed so that *C. glutamicum* can fulfill its potential [6, 7]. To achieve this, rapid and efficient genome editing methods suitable for *C. glutamicum* are needed.

Integrative plasmid vectors have been developed for gene deletion, mutation and insertion. These include suicide plasmids based on SacB, which hydrolyzes sucrose and synthesizes levan, leading to sucrose sensitivity in *C. glutamicum* [8, 9], and suicide plasmids based on the Cre/loxP system, in which Cre recombinase catalyzes specific recombination between two loxP sites [10, 11]. However, the efficiency of this gene deletion method is not very efficient because two rounds of homologous recombination are required and mutant selection after the second recombination is time-consuming [12, 13]. Therefore, a more efficient method for *C. glutamicum* genome editing is desirable.

The recent development of the CRISPR/Cas9 system provides a simple, sequence-specific platform for genome engineering [14, 15]. The widely used *Streptococcus pyogenes* (*S. pyogenes*) type II CRISPR/Cas9 system, which requires a mature CRISPR RNA (crRNA), a *trans*-activating CRISPR RNA (tracrRNA), and a DNA endonuclease, Cas9, has been harnessed for targeted genome editing in many organisms [15–17]. The Cas9 protein is an RNA-guided endonuclease that cleaves target DNA; a 20 bp complementary region (N20) within the crRNA can guide Cas9 to its specific target [18, 19]. The 20 nt sequence, known as the protospacer [20], contains a specific protospacer-adjacent motif (PAM) at its 3′ end [21]. The PAM sequence leads Cas9 to create a double-strand break (DSB) at the target sequence, and the DSB stimulates the DNA repair pathway by non-homologous end joining (NHEJ) or homolog-directed repair (HDR) [22–24]. The crRNA and tracrRNA can be fused together to generate a single synthetic guide RNA (sgRNA), which simplifies genome editing design [25].

The CRISPR/Cas9 system, has been widely applied in both prokaryotes and eukaryotes, such as *E. coli* [26–28], *Saccharomyces cerevisiae* [29], *Staphylococcus aureus* [25], *Bacillus subtilis* [30], *Lactococcal Phages* [17], higher-plants [31, 32], and animal cells [21, 33]. Moreover, the system has been used in *C. glutamicum* to manipulate the expression levels of specific genes [12], but not for genome editing, such as gene deletion or insertion, or the generation of point mutations. A *C. glutamicum* genome editing tool based on the CRISPR-Cpf1 system was recently reported; however, the authors of this study were unsuccessful in developing a CRISPR/Cas9-based system for use in *C. glutamicum* [34]. Here, we developed a CRISPR/Cas9-based genome editing method to investigate the function of *C. glutamicum* genes involved in recombinant protein expression. We analyzed the transcriptomes of *C. glutamicum* grown under different dissolved oxygen conditions to identify genes that might affect substance and energy metabolism and, therefore, might play important roles in the ability of *C. glutamicum* to express recombinant proteins [35]. We identified four genes, *porB*, *mepA*, *clpX*, and Ncgl0911, and used the system to disrupt them in *C. glutamicum* ATCC 13032 to investigate their endogenous functions and their effects on recombinant protein production. Highly efficient gene deletions were obtained via homolog-directed repair through the introduction of a DNA repair template. In addition, point mutations and gene insertions were achieved with an efficiency of 100 and 66.7%, respectively. We also expanded the system to *C. glutamicum* CGMCC1.15647, a host strain for recombinant protein production. Green fluorescent protein (GFP) was used as a model protein to examine the effect of different gene-deletion strains produced by the CRISPR/Cas9 system on recombinant protein expression and the results showed varying GFP expression levels in different strains. Overall, our CRISPR/Cas9-based genome editing method enabled rapid and efficient genome editing in a single step without the need for a marker gene, making this an effective tool for gene analysis and genome engineering in *C. glutamicum*.

## Results

### Construction of the CRISPR/Cas9 system in two plasmids

**Fig. 1:**
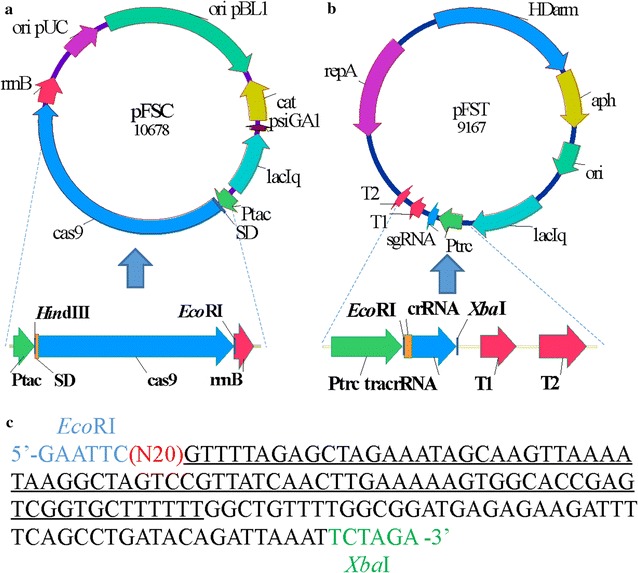
Design of the CRISPR/CAS9 system for gene deletion in *C. glutamicum*. **a** Strategy for construction of pFSC. Cas9 is controlled by the IPTG-inducible Ptac promoter, the SD sequence is designed to enhance the expression of Cas9; **b** strategy for construction of pFST. The sgRNA cassette is under the control of the IPTG-inducible Ptrc promoter, the 20 nt target sequence is shown in gold, the backbone is a temperature sensitive repA replicon, the HDarm is ligated into pFST at the *Bgl*II site; **c** strategy for construction of the sgRNA. The red N20 is the 20 nt target sequence, and the underlined sequences are the sgRNA scaffold. The *Eco*RI and *Xba*I sites are used to assemble the sgRNA into pFST

To establish a genome editing system in *C. glutamicum* based on CRISPR/Cas9, we designed and constructed a two-plasmid system that separated Cas9 and the sgRNA into pFSC and pFST plasmid series, respectively (Fig. 1a and b). pFSC was constructed from pXMJ19 [36], and included the Cas9 gene, an SD sequence and the Ptac promoter. We chose the strong Ptac promoter to drive the expression of Cas9, which is IPTG-inducible. The SD sequence (AAAGGAGGACAACTA) in front of the Cas9 gene ATG is indispensable for Cas9 protein expression. pFST was constructed in the temperature sensitive pEC-XK99E backbone [37] containing the temperature sensitive *repA* from pDTW109 [9], which enables fast curing of the plasmid after editing. An sgRNA containing an N20 sequence targeting the genomic locus of interest under control of the IPTG-inducible Ptrc promoter and a homologous repair template of the target gene were inserted into pEC-XK99E to give pFST. The homologous repair template regions upstream and downstream of the target locus were generated by PCR and assembled into pFST using an NEB Gibson assembly cloning kit. sgRNA was ligated into *Eco*RI and *Xba*I cloning sites of pFST, while the repair template was inserted into the *Bgl*II site. pFSC and pFST carry kanamycin and chloramphenicol resistance genes, respectively.

### Genome editing in *C. glutamicum* ATCC 13032 and *C. glutamicum* CGMCC1.15647

**Fig. 2:**
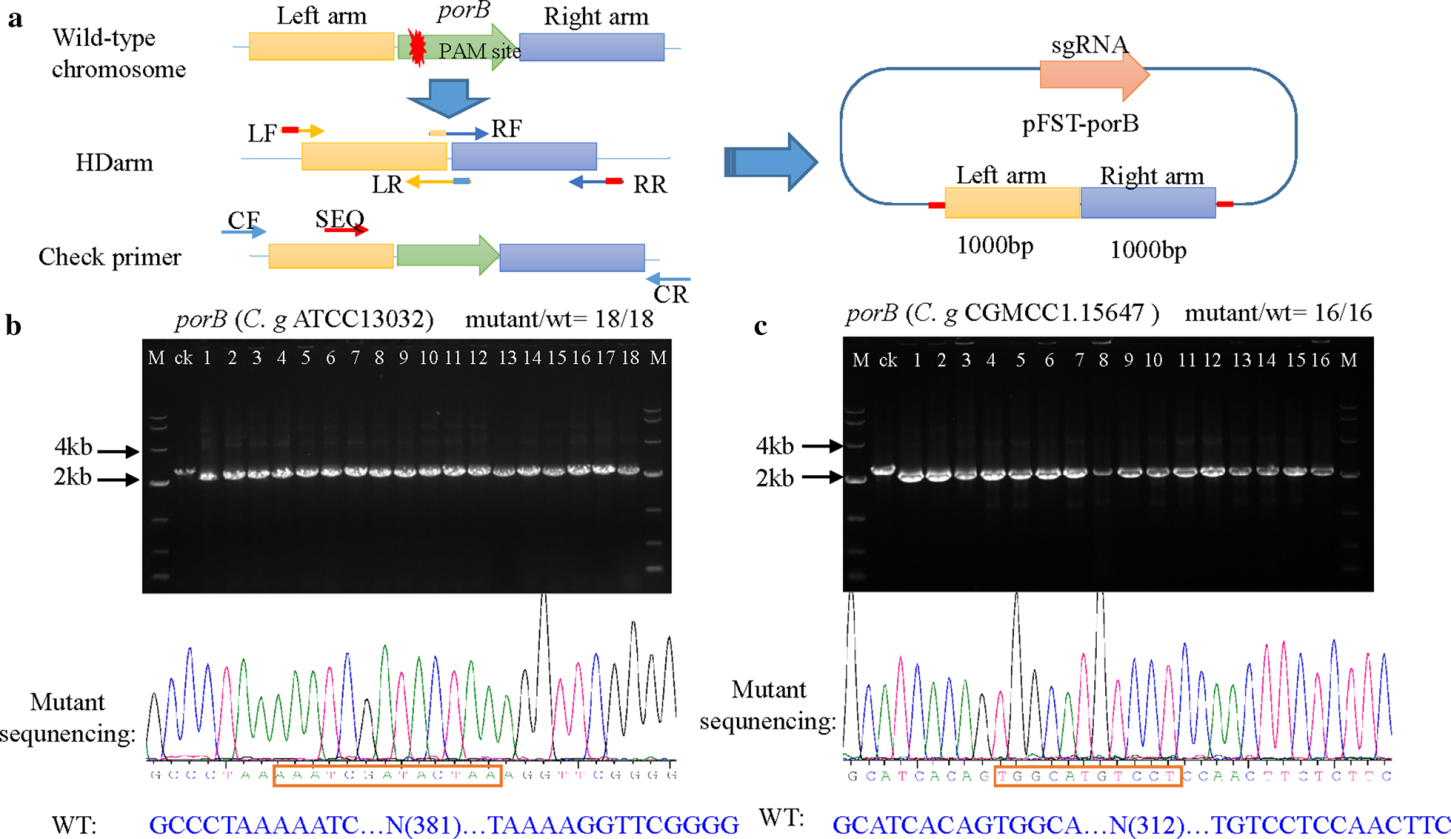
CRISPR/Cas9-mediated genome editing in *C. glutamicum* ATCC 13032 and *C. glutamicum* CGMCC1.15647. **a** Schematic depicting editing procedures. The left and right arms are regions from the targeted gene and are amplified by PCR from *C. glutamicum* genomic DNA. LF and LR primers are used to amplify the left arm, and RF and RR primers are used to amplify the right arm. For Gibson assembly, the 5′ end of LF contains a 20 bp overhang region of the 5′ end of the *Bgl*II site from the pFST plasmid. The 5′ end of LR contains a 10 bp overhang region of the 5′ end of the right arm. The 5′ end of RF contains a 10 bp overhang region of the 3′ end of the left arm. The 5′ end of RR contains a 20 bp overhang region of the 3′ end of the *Bgl*II site from the pFST plasmid. CF and CR are primers for PCR validation of editing efficiency. The SEQ primer is used for sequencing. **b** The CRISPR/Cas9 system mediated disruption of the *porB* gene in *C. glutamicum* ATCC 13032. The editing efficiency was 18/18. The lane ‘ck’ is the PCR product from the wild-type strain. These results were confirmed by sequencing. **c** The CRISPR/Cas9 system mediated disruption of the *proB* gene in *C. glutamicum* CGMCC1.15647. The editing efficiency was 16/16

To test the CRISPR/Cas9 system, the *porB* gene was chosen to be knocked out. This gene is an anion-selective channel that affects substance exchange in *C. glutamicum* [11, 38]. The sgRNA containing a 20 bp spacer was designed from the *porB* gene sequence and was checked to have no predicted off-target effects by BLAST searches against the *C. glutamicum* genomic sequence (https://blast.ncbi.nlm.nih.gov/Blast.cgi). The sgRNA sequence and the gene length are shown in Table 1. The sgRNA was subcloned into pFST, and after sequence validation, a correct plasmid named pFST-porBsgRNA was selected for transformation. First we transformed pFSC and pFST-porBsgRNA into *C. glutamicum* ATCC 13032. The sgRNA directed the Cas9 endonuclease to the *porB* locus, and the sgRNA/Cas9 complex generated a DSB at the locus. However, no colonies with the plasmid containing the sgRNA were observed after transformation, whereas more than 1 × 10^3^ colonies were obtained with the plasmid without the sgRNA (Additional file 1: Figure S1), indicating that the system containing only sgRNA and Cas9 was not effective in *C. glutamicum*. The DSB can be repaired by error prone NHEJ, but the efficiency of NHEJ in *C. glutamicum* is much lower than that in eukaryotic organisms. We, therefore, assembled the repair arms into pFST-porBsgRNA to assess editing efficiency by fixing the DSB through homolog-directed repair. In addition, three negative controls were used: the first was pFST-porB containing an sgRNA targeting the *porB* sequence and a homologous repair template, the second was pFST-porBT without an sgRNA, and the third was pFST-porBsgRNA without a homologous repair template. After transformation of the three plasmids, only the first produced DNA knock out. The plasmid not containing an sgRNA was wild-type, and no clones were obtained from the third plasmid because of the absence of the homology-directed repair template. As shown in Table 2 and Fig. 2b, the efficiency of deleting *porB* by this system was 100%, confirmed by both the PCR screening and sequencing.

**Table 1:** The PAM site and the sgRNA sequence used in this study

Gene	PAM sequence	PAM site and gene length	Target strand	GC (%)
*porB*	GGAGGATAGGTTTGCGAAGTCGG	83/381	NT	50
*mepA*	GGCACCT TCACCTCAGGATTCGG	214/576	T	55
*clpX*	GGCTGAAATCTCCGACGGCTTGG	194/1281	NT	60
Ncgl0911	GGTAACTGGGCTGGCCAAAAGGG	62/1260	NT	55
*mepA*1	GGTGGCGGTAGCGGTTGCGGTGG	62/576	NT	75
*mepA*2	GGATGCTGGTGCCATGGTGGCGG	77/576	NT	65
*mepA*3	GGAGGAAAGGCCTGCGTAGTCGG	108/576	NT	60
*mepA*4	GGCGGCTGGTGCGACGGCGGTGG	173/576	NT	85
*mepA*5	GGCCAGGAAATCGCAGGAATGGG	445/576	T	55
*mepA*6	GGCAGCCAAGGATTCTCCACCGG	467/576	T	60

The PAM sites are shown in underline. NT and T mean sgRNAs targeting to the template (T) or nontemplate (NT) respectively

**Table 2:** Results of the *porB* deletion in *C. glutamicum ATCC* 13032 *and C. glutamicum* CGMCC1.15647

No	Host cell	Plasmid	Element	Results (D/W/T)	Efficiency (%)
1	ATCC 13032	pFSC	cas9	0/5/5	0
2	ATCC 13032	pFST-porBsgRNA	sgRNA	0/5/5	0
3	ATCC 13032	pFST-porBT	Hdarm	0/5/5	0
4	ATCC 13032	pFSC, pFST-porBT	Cas9 + Hdarm	0/5/5	0
5	ATCC 13032	pFSC, pFST-porBsgRNA	Cas9 + sgRNA	0/0/0	0
6	ATCC 13032	pFST-porB	sgRNA + Hdarm	0/6/6	0
7	ATCC 13032	pFSC, pFST-porB	Cas9 + sgRNA + Hdarm (1000 bp)	18/0/18	100
8	CGMCC 1.15647	pFSC, pFST-porB15647	Cas9 + sgRNA + Hdarm (1000 bp)	16/0/16	100
9	ATCC 13032	pFSC, pFST-porB600	Cas9 + sgRNA + Hdarm (600 bp)	10/2/12	83.3
10	ATCC 13032	pFSC, pFST-porB300	Cas9 + sgRNA + Hdarm (300 bp)	10/2/12	83.3
11	ATCC 13032	pFSC, pFST-porB100	Cas9 + sgRNA + Hdarm (100 bp)	2/10/12	16.7

D, number of colonies that harbored gene-deleted cells; W, number of colonies that harbored wild type cells; T, total number of colonies used for PCR screening; Efficiency, probability of deletion events occurring, calculated as D/T * 100%; ATCC 13032, *C. glutamicum* ATCC 13032; CGMCC 1.15647, *C. glutamicum* CGMCC1.15647

To further assess the potential use of this system, we applied it to *C. glutamicum* CGMCC1.15647, a host strain for recombinant protein expression. As in *C. glutamicum* ATCC 13032, the efficiency was 100% (Fig. 2c). These observations indicated that the CRISPR/Cas9 system indeed deleted the *porB* gene in *C. glutamicum*.

### Different repair template fragments

**Fig. 3:**
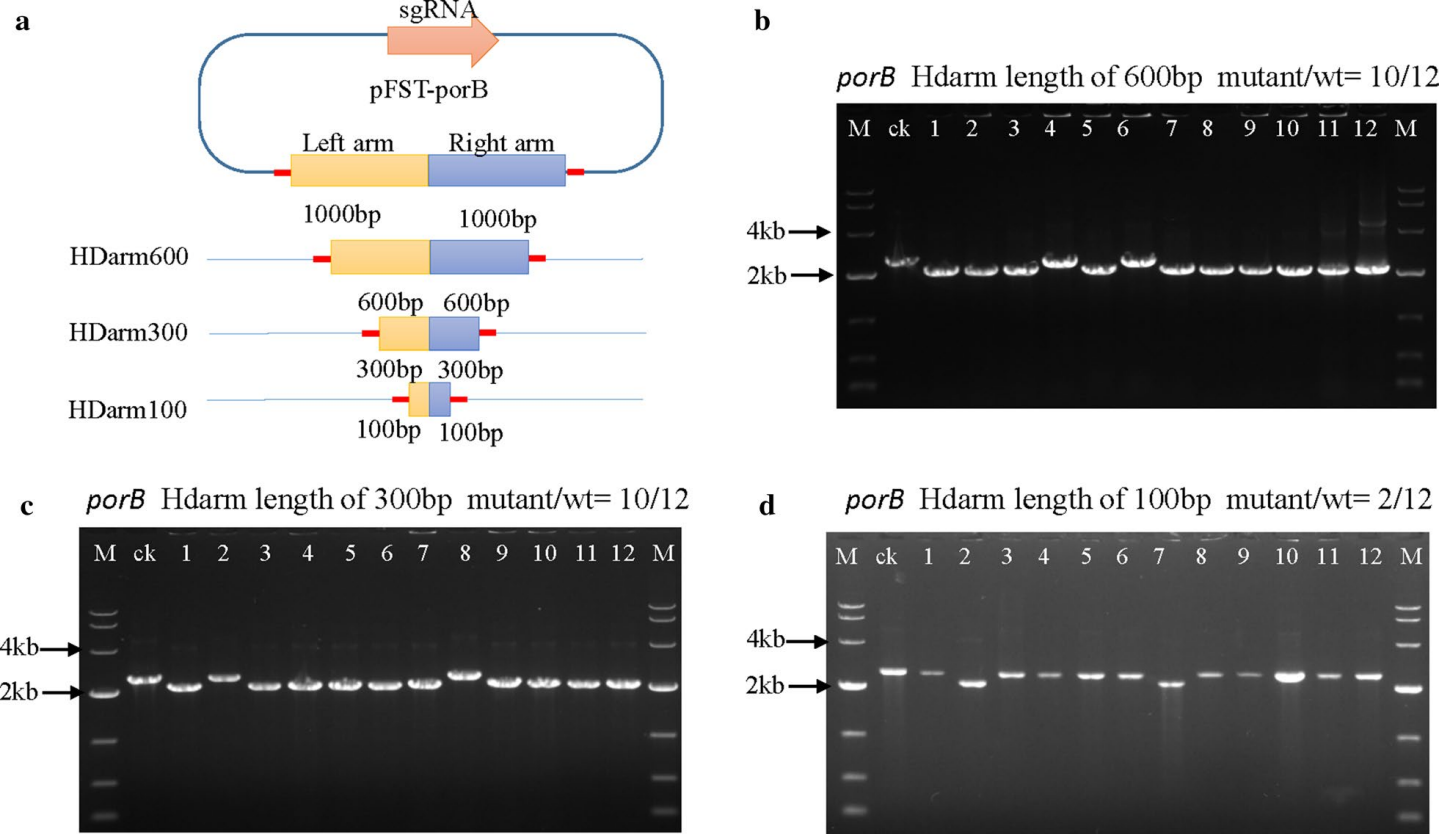
Evaluation of editing efficiency with different arm sizes. **a** Design of HDarms of various sizes (600, 300, 100 bp). Both sides of the HDarm contain a 20 bp overhang region of the *Bgl*II site from the pFST plasmid. **b** Disruption of the *porB* gene mediated by the CRISPR/Cas9 system in *C. glutamicum* ATCC 13032 with a 600 bp HDarm. The editing efficiency was 10/12, the lane ‘ck’ is the PCR product from the wild-type strain. **c** Disruption of the *porB* gene mediated by the CRISPR/Cas9 system in *C. glutamicum* ATCC 13032 with a 300 bp HDarm. The editing efficiency was 10/12. **d** Disruption of the *porB* gene mediated by the CRISPR/Cas9 system in *C. glutamicum* ATCC 13032 with a 100 bp HDarm. The editing efficiency was 2/12

To evaluate the effect of repair arm size on gene editing because the length of homologous arms affects recombination frequency. We constructed a series of donor templates with homologous arms of varied length (0.6, 0.3 and 0.1 kb) which could be easily synthesized and assembled. The vectors were constructed by assembling the repair arms into pFST-porBsgRNA and were then transformed into *C. glutamicum* ATCC 13032. Then we used PCR and sequence analysis to validate the deletions. As shown in Table 2 and Fig. 3, efficiency with the 0.3 and 0.6 kb arms was 83.3%, which is lower than the efficiency with 1 kb arms. However, the efficiency with 0.1 kb arms was only 16.7%.

### Editing different genes

**Fig. 4:**
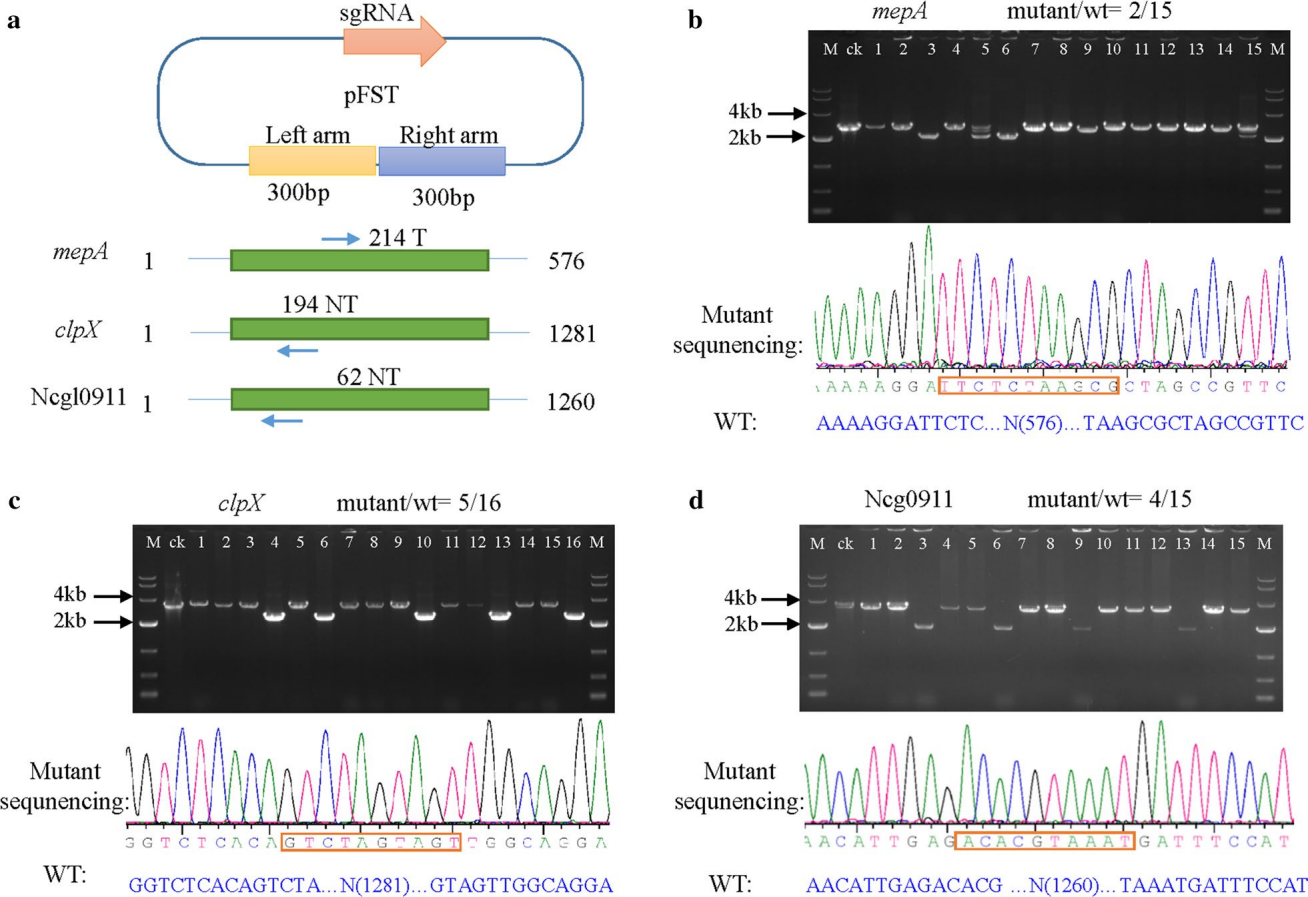
Genome editing mediated by CRISPR/Cas9 in *C. glutamicum* ATCC 13032. **a** Schematic depicting editing procedures, the PAM site and gene length. **b** Disruption of the *mepA* gene mediated by the CRISPR/Cas9 system in *C. glutamicum* ATCC 13032. The editing efficiency was 2/15. The lane ‘ck’ is the PCR product from the wild-type strain. **c** Disruption of the *clpX* gene mediated by the CRISPR/Cas9 system in *C. glutamicum* ATCC 13032. The editing efficiency was 5/16. **d** Disruption of the Ncgl0911 gene mediated by the CRISPR/Cas9 system in *C. glutamicum* ATCC 13032. The editing efficiency was 4/15

To further validate the system, three other *C. glutamicum* genes were targeted. These were *mepA*, *clpX* and Ncgl0911, which encode genes involved in cell wall metabolism, proteolysis and the two-component system, respectively [39–42]. We constructed the knockout plasmids, pFST-mepA, pFST-clpX and pFST-0911, and used them to disrupt *mepA*, *clpX* and Ncgl0911 genes using the above CRISPR/Cas9 method. The proportion of mutants in the transformants was determined by PCR and sequencing. The PAM site and the gene length are shown in Table 1. We showed above that 300 bp repair arms are sufficient for gene deletion; therefore, we amplified 300 bp left and right arms separately by PCR and assembled them into pFST (Fig. 4a). For *mepA*, two pure mutant and two mixed mutant and wild-type populations were observed from 15 colonies (Fig. 4b). In contrast, no mixed colonies were detected for *clpX* and Ncgl0911 and the mutation efficiency was 5/16 and 4/15, respectively (Fig. 4c and d). These results demonstrated the high efficiency of this system for gene deletion in *C. glutamicum* ATCC 13032.

### Point mutation and gene insertion in *C. glutamicum*

**Fig. 5:**
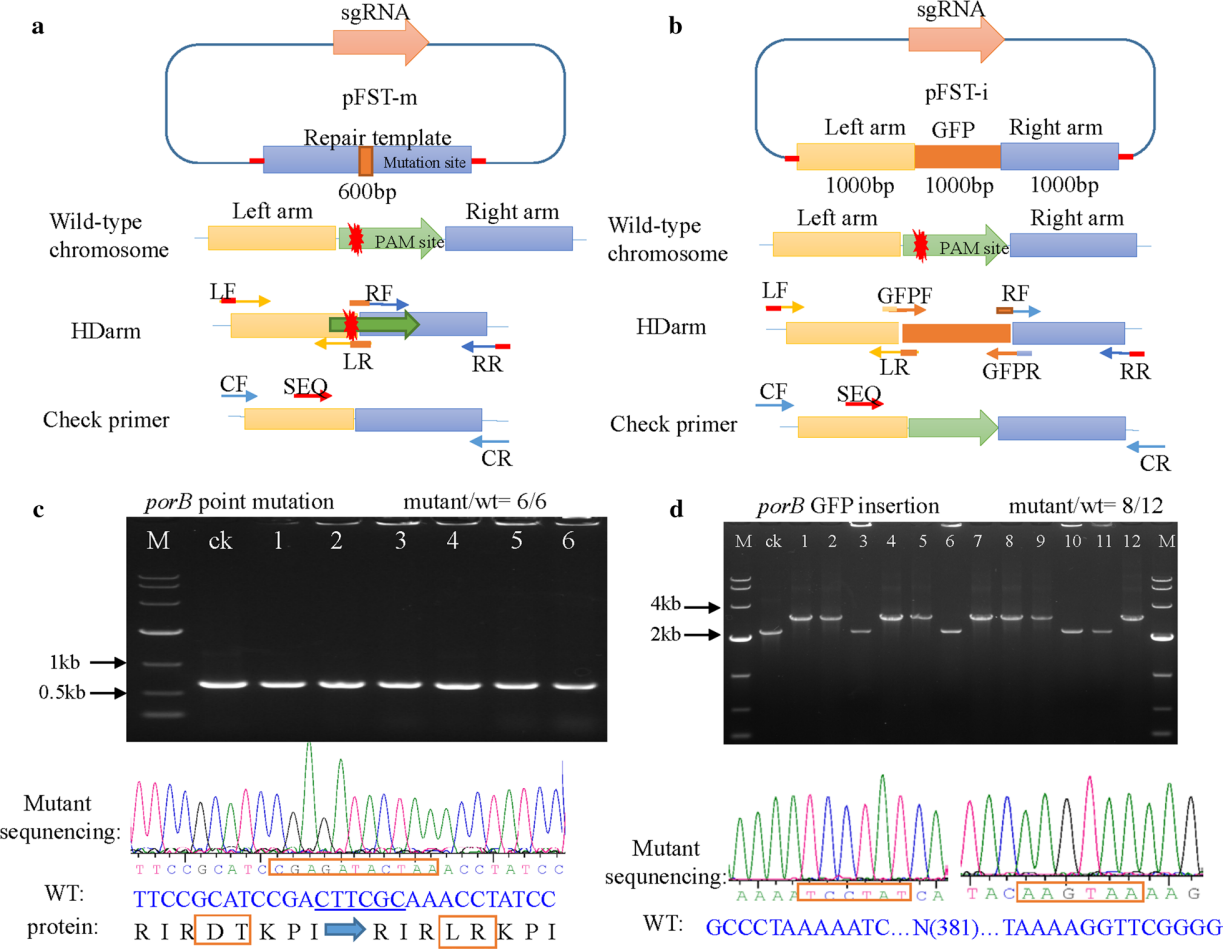
Point mutation and gene insertion mediated by the CRISPR/Cas9 system in *C. glutamicum* ATCC 13032. **a** Schematic depicting the procedure for generating point mutations. The 5′ end of LR contains a 10 bp overhang region of the 5′ end of the right arm. The 5′ end of RF contains a 10 bp overhang region of the 3′ end of the left arm. The point mutation site was designed in the primers FR and RF. CF and CR are primers for PCR validation of editing efficiency. The SEQ primer is used for sequencing. **b** Schematic depicting the procedure for gene insertion. The 5′ end of the LR contains a 10 bp overhang region of the 5′ end of GFP. The 5′ end of GFPF contains a 10 bp overhang region of the 3′ end of the left arm. The 5′ end of GFPR contains a 10 bp overhang region of the 5′ end of the right arm. The 5′ end of RF contains a 10 bp overhang region of the 3′ end of the GFP arm. **c** The point mutation mediated by CRISPR/Cas9 in *C. glutamicum* ATCC 13032. The mutation efficiency was 6/6 and was confirmed by sequencing. **d** Gene insertion mediated by CRISPR/Cas9 in *C. glutamicum* ATCC 13032. The *porB* gene was replaced by the *gfp* gene. The efficiency for *gfp* insertion was 8/12, confirmed by PCR and sequencing

The study of gene functions can often be facilitated by the generation of point mutations in a target gene. We, therefore, designed this system to mutate specific sites in the genome. We first created a six base mutagenic site in the repair template and assembled it into pFST-m (Fig. 5a). Next, we transformed the plasmid into *C. glutamicum* ATCC 13032. We then used PCR and sequencing to validate the mutation. A high editing efficiency of 6/6 was observed (Fig. 5c).

Analysis of gene functions and pathways may also need the insertion of gene(s) into the genome, such as a reporter system to monitor the activity of a promoter of interest. To test this system for creating insertion mutants, we assembled the GFP gene (*gfp*) and repair arms into pFST-i (Fig. 5b). The repair arm length was 1000 bp. After transforming the plasmid into *C. glutamicum* ATCC 13032, we observed a high editing efficiency of 8/12 (Fig. 5d). The efficiency of gene insertion for 300 bp repair arms was not as high as that for 1000 bp arms at only 3/12, possibly owning to the insert gene being longer than the repair arms (Additional file 1: Figure S2).

### Effect of different sgRNAs on editing efficiencies

The efficiency of editing the *mepA* gene was not as high as that for other genes, showing that gene editing efficiencies may be affected by different sgRNAs targeting different sites and different strands of the same gene. To improve efficiency, we tested different sgRNAs targeting different sites and different strands of the *mepA* gene. The PAM site and the sgRNA sequence are shown in Table 1. After transformation of the plasmid into *C. glutamicum* ATCC 13032, we used PCR and sequencing to validate the deletion. As shown in Table 3 and Additional file 1: Figure S3, the efficiencies of sgRNAs 1–6 were 3/12, 0/12, 10/12, 4/12, 12/12 and 6/12, respectively. Repair arms of the same length but binding to different sgRNAs were used and generated different editing efficiencies, increased the efficiencies from 13.3 to 100%. These results indicated that different sgRNAs have a great influence on editing efficiency and that it is better to use an sgRNA with the GC content under 60%.

**Table 3:** Results of the *mepA* deletion in *C. glutamicum* ATCC 13032

Plasmid	Element	Results (D/W/T)	Efficiency (%)
pFSC, pFST-mepA1	Cas9 + sgRNA1 + Hdarm (300 bp)	3/9/12	25
pFSC, pFST-mepA2	Cas9 + sgRNA2 + Hdarm (300 bp)	0/12/12	0
pFSC, pFST-mepA3	Cas9 + sgRNA3 + Hdarm (300 bp)	10/2/12	83.3
pFSC, pFST-mepA4	Cas9 + sgRNA4 + Hdarm (300 bp)	4/8/12	33.3
pFSC, pFST-mepA5	Cas9 + sgRNA5 + Hdarm (300 bp)	12/0/12	100
pFSC, pFST-mepA6	Cas9 + sgRNA6 + Hdarm (300 bp)	6/6/12	50

D, number of colonies that harbored gene-deleted cells; W, number of colonies that harbored wild type cells; T, total number of colonies used for PCR screening; Efficiency, probability of deletion events occurring, calculated as D/T * 100%

### Off target analysis of edited strains

To analysis the off target effect in *C. glutamicum* after gene editing by CRISPR/Cas9 system, genome re-sequencing were performed to identify all the single nucleotide polymorphism (SNP) and insertions and deletions (Indel) using the *proB*-deleted strain and the *mepA*-deleted strain with wild type *C. glutamicum* ATCC13032 as the negative control. In addition, to analysis whether the Cas9 protein create the off target without the sgRNA, the SNP and Indel of the wild type strain containing Cas9 protein were also detected. Result showed no off-target mutations were detected in the wild type strain containing Cas9 protein, that is, compared with the wild type the SNP and Indel were not detected in this strain. Meanwhile, in the *mepA*-deleted strain no SNP and Indel were detected, while in the *porB*-deleted strain no SNP but 1 Indel with 1 base deleted was identified (Additional file 1: Table S3). The results suggested the CRISPR/Cas9 gene editing system with HDR can generate the off target in a very low probability.

### Quantification of GFP activity

**Fig. 6:**
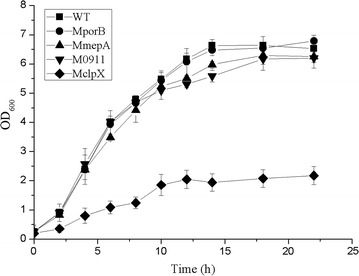
Growth phenotypes of the *porB*, *mepA*, *clpX* and Ncgl0911-deleted strains. WT, wild-type strain; MporB, *porB*-deleted mutant; MmepA, *mepA*-deleted mutant; M0911, Ncgl0911-deleted mutant; MclpX, *clpX*-deleted mutant; the *clpX* mutant showed impaired growth compared with the wild-type strain while the other mutants showed the same growth rate as the wild-type. Date are representative of triplicate cultures

**Fig. 7:**
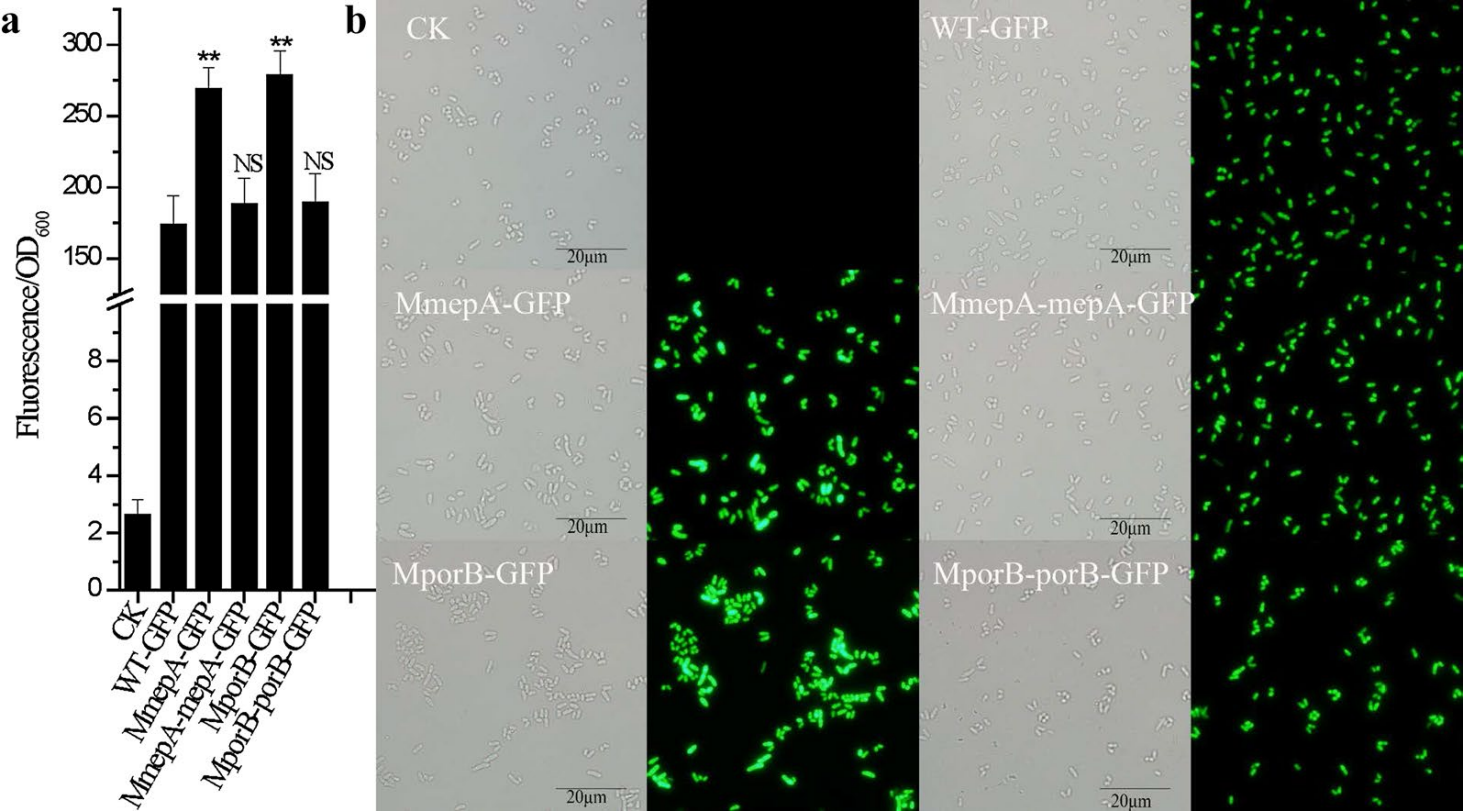
GFP expression in the *porB*, *mepA*, *clpX* and Ncgl0911-deleted strains. CK is a negative control of the wild-type strain containing pXMJ19 without the *gfp* gene. WT-GFP is a positive control of the wild-type strain containing pXMJ19-EGFP; MmepA-GFP is the *mepA*-deleted mutant with plasmid pXMJ19-EGFP; MmepA-mepA-GFP is the *mepA*-deleted mutant complemented by *mepA* gene and containing pXMJ19-EGFP; MporB-GFP is the *porB*-deleted mutant with plasmid pXMJ19-EGFP; MporB-porB-GFP is the *porB*-deleted mutant complemented by *porB* gene and containing pXMJ19-EGFP. **a** Fluorescence intensities normalized against culture OD_600_ were used to indicate the expression of GFP. **b** The expression of GFP was determined by fluorescence microscopy with an exposure time of 1 s. The *mepA* and *porB* mutants showed GFP expression enhanced by 55.2 and 62.4%, respectively, relative to the wild type

The four *C. glutamicum* genes, *porB*, *mepA*, *clpX* and Ncgl0911, encoding genes involved in cell wall metabolism, anion absorption, proteolysis, and the two-component system, were identified as differentially expressed under different dissolved oxygen levels by the analysis of transcriptome data (Accession Number GSE77502). Dissolved oxygen is an important factor that significantly influences metabolism and recombinant protein product yield in *C. glutamicum* when cultured in a bioreactor [35]. We, therefore, speculated that these genes play important roles in affecting recombinant protein expression in *C. glutamicum*. In our study, GFP was chosen as a model protein to evaluate the gene deletion strains. We removed the *lacIq* gene from pXMJ19 to create a constitutive expression vector. We then constructed the GFP expression plasmid, pXMJ19-EGFP, by introducing the *gfp* gene into pXMJ19 (Additional file 1: Figure S4). The phenotype of the generated mutants was confirmed by genetic complementation, here, we used the plasmid pECXK99 as a borne to complement the deleted gene (Additional file 1: Figure S5). After transforming the plasmid into *C. glutamicum* ATCC 13032, the gene deletion strains and the complementation strains, we cultured the different strains and measured their OD_600_ values and fluorescence intensities. The results indicated impaired growth of the *clpX* mutant compared with the wild-type strain, while the other mutant strains showed the same growth rate as the wild type (Fig. 6). The complementation strains of *porB*, *mepA* and Ncgl0911 showed the same growth rate as the wild type and mutant strains (Additional file 1: Figure S6). The *clpx* gene deletion strain can’t survival in the medium for competent cell, so we can’t get the complementation strain of *clpx* gene. The *mepA* and *porB* mutants showed 55.2 and 62.4% greater GFP fluorescence respectively, compared with wild type (Fig. 7, Additional file 1: Figure S7), but the reason for this requires further study. This CRISPR/Cas9 system provides an efficient way to study the function of different genes and identified a key gene involved in recombinant protein expression.

## Discussion

In this study, we adapted the CRISPR/Cas9 system from *S. pyogens* to be efficient at generating gene deletions, point mutations and gene insertions in *C. glutamicum* ATCC 13032 and *C. glutamicum* CGMCC1.15647. Compared with published genome modification methods, methods based on the CRISPR/Cas9 system can fast and accurate achieve genome editing.

In this study we observed that *C. glutamicum* cannot survive after the DNA DSBS introduced by Cas9 in the absence of a homologous recombination template. Because the efficiency of NHEJ in *C. glutamicum* is much lower than that in eukaryotic organisms (Additional file 1: Figure S1). So a homologous recombination template is necessary in this system [13]. We observed nearly 100% efficiency for *porb* gene deletion in *C. glutamicum* ATCC 13032 and *C. glutamicum* CGMCC1.15647 when a homology-based repair template was supplied. The system is readily applicable to other related *Corynebacteria*.

Typically, relatively long flanking regions of at least 700 bp are used for incorporation of novel genetic elements into the *C. glutamicum* genome [27]. A powerful genome editing tool should have a high efficiency and be independent of a marker gene. Long homologous arms may introduce unwanted mutations and increase the difficulty of plasmid construction. An arm length of greater than 0.3 kb gave a very high editing efficiency. Longer repair arms can increase the efficiency of gene editing but 300 bp repair arms are long enough for genome deletion.

By assembling the *gfp* gene into *porB* repair arms we also used this system to create a *gfp* inserted mutation. The efficiency of gene insertion for 300 bp repair arms was not as high as that for 1000 bp arms (Additional file 1: Figure S2), possibly owning to the insert gene being longer than the repair arms. This low efficiency may be related to low homologous recombination efficiency. It is, therefore, better for the repair arms to be longer than the target gene for gene insertion.

In addition, it is difficult to express some high molecular weight proteins in *C. glutamicum*; therefore, we codon-optimized the *Cas9* gene and an SD sequence was added in front of the ATG initiation codon. We found that no transformants were produced by the plasmid containing the de-repressed Ptac promoter. We speculate that Cas9 is toxic to *C. glutamicum*; therefore, we used the inducible Ptac promoter and a low concentration of IPTG to induce expression of Cas9. Jiang et al. [34] also found that Cas9 was toxic to *C. glutamicum* and they speculated that SpCas9 binds tightly to PAMs, even without a crRNA. In our study, the SNP and Indel were not detected in the *C. glutamicum* wild type strain containing the Cas9 without sgRNA compared with the wild type strain, indicating the toxic was not caused by the off target. We also found the transformants containing the Cas9 plasmid grew at a lower rate than the wild type. When the plasmid was cured, the growth rate of strains returned to the normal levels. Moreover, the Cas9 plasmid is easy to be lost without antibiotics. Therefore we can use this effect to cure the plasmid after performing gene editing.

In summary, the CRISPR/Cas9 system developed in this study will accelerate research into gene function, metabolic engineering, specific biosynthetic pathway analysis, and genetic modification for recombinant protein expression.

## Methods

### Strains, plasmids, media and reagents

All bacterial strains and plasmids used in this study are described in Additional file 1: Table S1. *E. coli* DH5α was used as a cloning host for plasmid construction. The replicon initiator of pEC-XK99E was replaced with the temperature sensitive *repA*, which is from PDTW109 [9]. *C. glutamicum* ATCC 13032 was purchased from the American Type Culture Collection (ATCC). *C. glutamicum* CGMCC1.15647 was used as the host for foreign protein expression and was donated by Zhangjiagang Huachang Pharmaceutical Co. (Zhangjiagang City, China).

*Escherichia coli*, were cultured in LB medium (Luria–Bertani: 10 g/L tryptone, 5 g/L yeast extract and 10 g/L NaCl) at 37 °C with shaking at 200 rpm. *C. glutamicum* was cultured in LBB medium (LB supplemented with brain heart infusion: 10 g/L tryptone, 5 g/L yeast extract, 10 g/L brain heart infusion Broth and 10 g/L NaCl) at 30 °C with shaking at 200 rpm. LBHIS (LB supplemented with brain heart infusion and sorbitol: 5 g/L tryptone, 2.5 g/L yeast extract, 18.5 g/L brain heart infusion Broth, 91 g/L sorbitol and 5 g/L NaCl) medium was used for obtaining transformants of *C. glutamicum*. Antibiotics were added at the following concentrations: in *E. coli*, kanamycin 30 μg/mL, ampicillin 100 μg/mL, and chloramphenicol 30 μg/mL; in *C. glutamicum*, kanamycin 10 μg/mL, and chloramphenicol 10 μg/mL.

Plasmid DNA was extracted using an AxyPrep Plasmid Miniprep kit (Axygen, Union City, CA, USA). DNA fragments from polymerase chain reactions (PCRs) and restriction enzyme digestions were purified using the AxyPrep Gel Extraction Kit (Axygen, Union City, CA, USA). Genomic DNA was extracted using a Bacterial genomic DNA Extraction Kit (TIANGEN, Beijing, China). Taq polymerase and T4 DNA ligase were purchased from Takara (Dalian, China). Restriction endonucleases were purchased from Thermo scientific (San Jose, CA, USA). Q5 and Gibson assembly kits were purchased from NEB (Beverly, MA, USA). Primers were purchased from Genweiz (Suzhou, China).

### sgRNA design

The sgRNA candidate sequences target PAM recognition domains in the genome, and all candidate sgRNA-target sequences had N20NGG motifs. Target gene sequences were downloaded from NCBI GenBank and analyzed by Vector NTI software (Thermo Fisher Scientific) for selection of sgRNA PAM sites using GN19NGG as a motif. The 3′ ends of protospacers avoided the sequence TTTT, and the GC content was between 40 and 60%. All protospacer candidates were blast searched against the NCBI *C. glutamicum* reference genome to identify sgRNA off-target sites that might produce off-target effects (https://blast.ncbi.nlm.nih.gov/Blast.cgi, Reference Sequence: NC_003450.3). All the sgRNA sequences (N20 sequences) and their PAM sites used are given in Table 1. Primers used are given in Additional file 1: Table S2.

### Plasmid construction

The two-plasmid system, in which Cas9 and sgRNA are in separate plasmids, pFSC and pFST, was used for genome editing as shown in Fig. 1a and b. The Cas9 used in our system was codon-optimized for *C. glutamicum* using the Genweiz Sequence Analysis program to give a GC content of 52% compared with 35% in *S. pyogens*. The construction procedure of the pFSC plasmid was as follows: the codon-optimized *cas9* gene was PCR-amplified from plasmid pcas9. The SD sequence, which is indispensable for Cas9 expression, was contained in the primer. Then the fragment was subcloned into pXMJ19 using *Hin*dIII and *Eco*RI sites, to be under the control of the IPTG inducible Ptac promoter.

The temperature sensitive plasmid, pEC-XK99E, was selected as the backbone of pFST. The 20 nt target sequence within the sgRNA scaffold was amplified from psgRNA (Fig. 1c). The sgRNA scaffold was flanked by *Eco*RI and *Xba*I restriction sites to allow easy insertion into pFST. sgRNA expression was inducible by IPTG under the control of the Ptrc promoter.

Left and right repair arms, from the 5′ and 3′ regions of the targeted genes, respectively, were amplified by PCR form *C. glutamicum* genomic DNA. The left arm was amplified by primers LF and LR, the right arm was amplified by primers RF and RR. The outer two primers (LF and RR) contained 20 bp overhang regions of the 5′ and 3′ ends of the *Bgl*II site from the pFST plasmid, respectively, and the inner two primers (FR and RF) contained 10 bp overhang regions of the other repair arm (Fig. 2a). After gel purification of the fragments and the *Bgl*II-digested pFST plasmid, the left arm, right arm and plasmid were assembled using a Gibson assembly cloning kit (NEB). The construction of the pFST plasmid was verified by PCR using the primers pecBglF and pecBglR, and was confirmed by sequencing.

The construction of repair arms used to generate point mutations and gene insertions was the same as that of gene deletion. The mutation site was designed in the inner two primers. The insertion gene primers contained 10 bp overhang regions of the left and right repair arms and arms were assembled using the Gibson assembly cloning kit (Fig. 4a, b).

### Genome editing

*Corynebacterium glutamicum* competent cells were prepared using a previously reported method [9]. Electroporation was performed in a 1 mm gene pulser cuvette (Bio-Rad, USA) at 1.8 kV, Plasmids were added to 100 μL competent cells that had been thawed on ice for 5 min and mixed gently to ensure even distribution. After electroporation, 1 mL LBHIS medium was immediately added to the cells he cell suspension and incubated for 6 min at 46 °C without shaking. The cells were incubated at 30 °C for 2 h, spread onto LBHIS agar containing kanamycin (10 μg/mL), chloramphenicol (10 μg/mL) and IPTG (0.01 mM), and then incubated 18 h at 30 °C. Transformants were confirmed by PCR amplification of the *cas9* gene and the repair arm. To obtain the gene-deletion strain directly, 1 μg pFSC and pFST plasmids were coelectroporated, but the transformation efficiency was low. To increase the transformation efficiency, competent cells containing the pFSC plasmid can be obtained first, and then used for other round of transformation.

### Re-sequencing analysis

Re-sequencing was performed to detect the off target in edited strains. Total DNA was extracted from *C. glutamicum* according to manufacturer’s protocol (TIANGEN, Beijing, China). DNA quality was determined using Qubit Fluorometer (Thermo Fisher Scientific, San Jose, CA, USA) to determine total mass and Fragment Analyzer to determine DNA integrity. The genome of *C. glutamicum* was sequenced using an Illumina HiSeq 4000 system (Illumina, San Diego, CA, USA) at the Beijing Genomics Institute (Shenzhen, China). Genomic DNA was sheared randomly to construct three read libraries with lengths of (300 bp) by a Bioruptor ultrasonicator (Diagenode, Denville, NJ, USA) and physico-chemical methods. The paired-end fragment libraries were sequenced according to the Illumina HiSeq 4000 system’s protocol. Raw reads of low quality from paired-end sequencing (those with consecutive bases covered by fewer than five reads) were discarded. The sequenced reads were assembled using SOAPdenovo v1.05 software.

### Plasmid curing

To cure mutant strains of the pFST plasmid to enable their use in a second round of genome editing, the mutant strains were inoculated in 5 mL of LBB medium containing chloramphenicol (10 μg/mL). The culture was incubated at 30 °C overnight and the next day 50 μL of culture was diluted 1:100 in 5 mL LBB, and incubated at 37 °C until the culture had visibly grown. Then cells were streaked onto an LBB plate and cultured overnight at 30 °C. Colonies cured of knockout plasmid were confirmed by streaking them onto LBB plates containing kanamycin and by PCR analysis.

For curing edited clones of pFSC, colonies harboring pFSC were inoculated into 5 mL LBB medium and grown at 30 °C to an OD_600_ of 1.0, and then 5 μL IPTG (100 mM/mL) was added. The culture was incubated overnight and streaked onto LBB plates without antibiotics and IPTG. The colonies were confirmed as cured by streaking them onto LBB plates containing chloramphenicol and by PCR analysis.

### Detection of GFP activity

To evaluate recombinant protein expression in *porB*, *mepA*, *clpX* and Ncgl0911 knock-out strains, we constructed a GFP expression plasmid, pXMJ19-EGFP, as follows. The GFP gene was amplified by PCR from a pEGFP-N1 template. The amplified fragment was ligated into *Hin*dIII and *Bam*HI sites of pXMJ19. The *lacIq* gene was removed from pXMJ19 to make it a constitutive expression vector. The physical map of pXMJ19-EGFP is shown in Additional file 1: Figure S5. The resulting plasmid, pXMJ19-EGFP, was introduced into the four gene-deleted *C. glutamicum* ATCC 13032 strains. To check the generated mutants by genetic complementation, plasmid pECXK99 was used as borne to expression the deleted gene. The *lacIq* gene was removed from pECXK99 to make it a constitutive expression vector. The deleted gene was amplified from the chromosome and was ligated into pECXK99. The physical map of pECXK99-gene is shown in Additional file 1: Figure S5. The GFP expression plasmid, pXMJ19-EGFP, was introduced into the complementation strains. These were then grown overnight in 24-well deep-well plates (LifeFeng, Hangzhou, China) containing 2 mL culture medium per well. Cultures were then diluted 1:100 into 2 mL fresh medium and grown for 24 h. The OD_600_ and fluorescence intensity values were measured by a Synergy H4 microplate reader (BioTek, USA). The excitation of EGFP was at 488 nm and emission was at 507 nm. Cultivation and measurements were performed in triplicate. Fluorescence intensities normalized against culture OD_600_ were used to indicate the expression level of GFP. Fluorescence intensity was also analyzed by fluorescence microscopy (OLYMPUS) with an exposure time of 1 s. GFP protein was also detected by 12% (w/v) sodium dodecyl sulfate-polyacrylamide gel electrophoresis (SDS-PAGE).

## Additional file

**Additional file 1.** Additional Figures S1–S7 and Tables S1–S3.

## Abbreviations

crRNA: CRISPR RNA
tracrRNA: *trans*-activating CRISPR RNA
PAM: protospacer-adjacent motif
DSB: double-strand break
NHEJ: non-homologous end joining
HDR: homolog-directed repair
sgRNA: synthetic guide RNA
GFP: green fluorescent protein
SNP: single nucleotide polymorphism
Indel: insertions and deletion
