# Supplementary material for: Efficient gene editing in Corynebacterium glutamicum using the CRISPR/Cas9 system
Source: Microb Cell Fact. 2017 Nov 14;16:201. doi: 10.1186/s12934-017-0814-6 (PMC5686833; doi:10.1186/s12934-017-0814-6)
Supplement: Supplementary file 1 — Additional file 1. Additional Figures S1–S7 and Tables S1–S3. [file 12934_2017_814_MOESM1_ESM.docx]

Additional information

**Efficient gene editing in *Corynebacterium glutamicum* using the CRISPR/Cas9 system**

Feng Peng ^1,2,3^, Xinyue Wang ^1,2,3^, Yang Sun ^1,2,3^, Guibin Dong ^1,2,3^, Yankun Yang ^1,2,3^, Xiuxia Liu ^1,2,3*^, Zhonghu Bai ^1,2,3*^

1. National Engineering Laboratory for Cereal Fermentation Technology, Jiangnan University, Wuxi 214122, China.

2. The Key Laboratory of Industrial Biotechnology, Ministry of Education, School of Biotechnology, Jiangnan University, Wuxi 214122, China.

3. The Key Laboratory of Carbohydrate Chemistry and Biotechnology, Ministry of Education, School of Biotechnology, Jiangnan University, Wuxi 214122, China

Table of contents

[Figure S1 2](#_Toc496879350)

[Figure S2 3](#_Toc496879351)

[Figure S3 4](#_Toc496879352)

[Figure S4 5](#_Toc496879353)

[Figure S5 6](#_Toc496879354)

[Figure S6 7](#_Toc496879355)

[Figure S7 8](#_Toc496879356)

[Table S1 9](#_Toc496879357)

[Table S2 11](#_Toc496879358)

[Table S3 15](#_Toc496879359)

[References 16](#_Toc496879360)

Figure S1. Effects of cas9, targeting sgRNA, and HD repair arm in the CRISPR/Cas9 two plasmid system. The lane of ck is the PCR Product from the wild-type. 1-7: The effects of clonies without cas9, sgRNA or the HD repair arm, the plasmid used as the table.


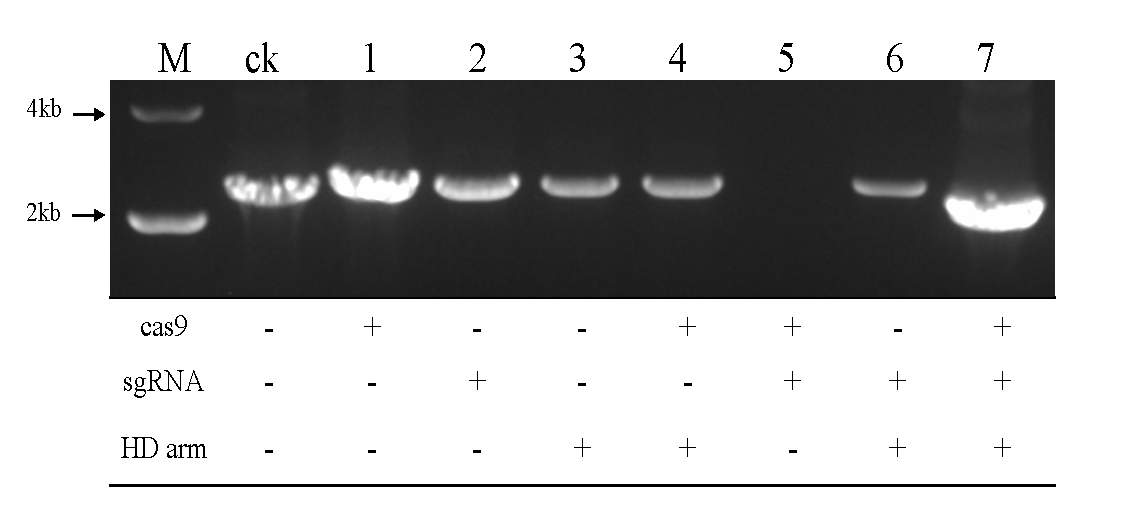


Figure S2. Gene insertion mediated by CRISPR/Cas9 system in the *C. glutamicum* ATCC 13032. (a) Schematic depicting the procedure for gene insertion. The length of left arm and right arm are 300bp. (b) Gene insertion mediated by CRISPR/Cas9 in the *C. glutamicum* ATCC13032. The efficiency for *gfp* insertion was 3/12, confirmed by PCR and sequencing, the lane ‘ck’ is the PCR product from the wild-type strain.


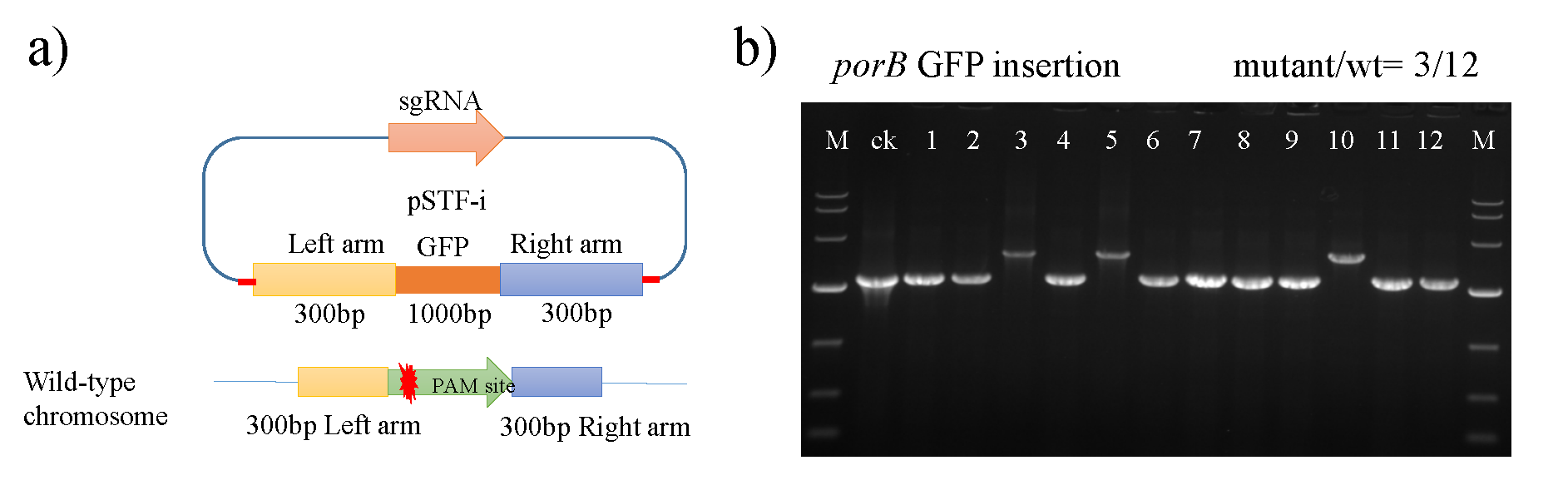


Figure S3. The results of the *mepA* deletion in *C. glutamicum* ATCC 13032 with diffenent sgRNA. (a) Disruption of the *mepA* gene mediated by sgRNA *mepA*1. The editing efficiency was 3/12, confirmed by PCR and sequencing the lane ‘ck’ is the PCR product from the wild-type strain. (b) Disruption of the *mepA* gene mediated by sgRNA *mepA*2. The editing efficiency was 0/12. (c) Disruption of the *mepA* gene mediated by sgRNA *mepA*3. The editing efficiency was 10/12. (d) Disruption of the *mepA* gene mediated by sgRNA *mepA*4. The editing efficiency was 4/12. (e) Disruption of the *mepA* gene mediated by sgRNA *mepA*5. The editing efficiency was 12/12. (f) Disruption of the *mepA* gene mediated by sgRNA *mepA*6. The editing efficiency was 6/12.


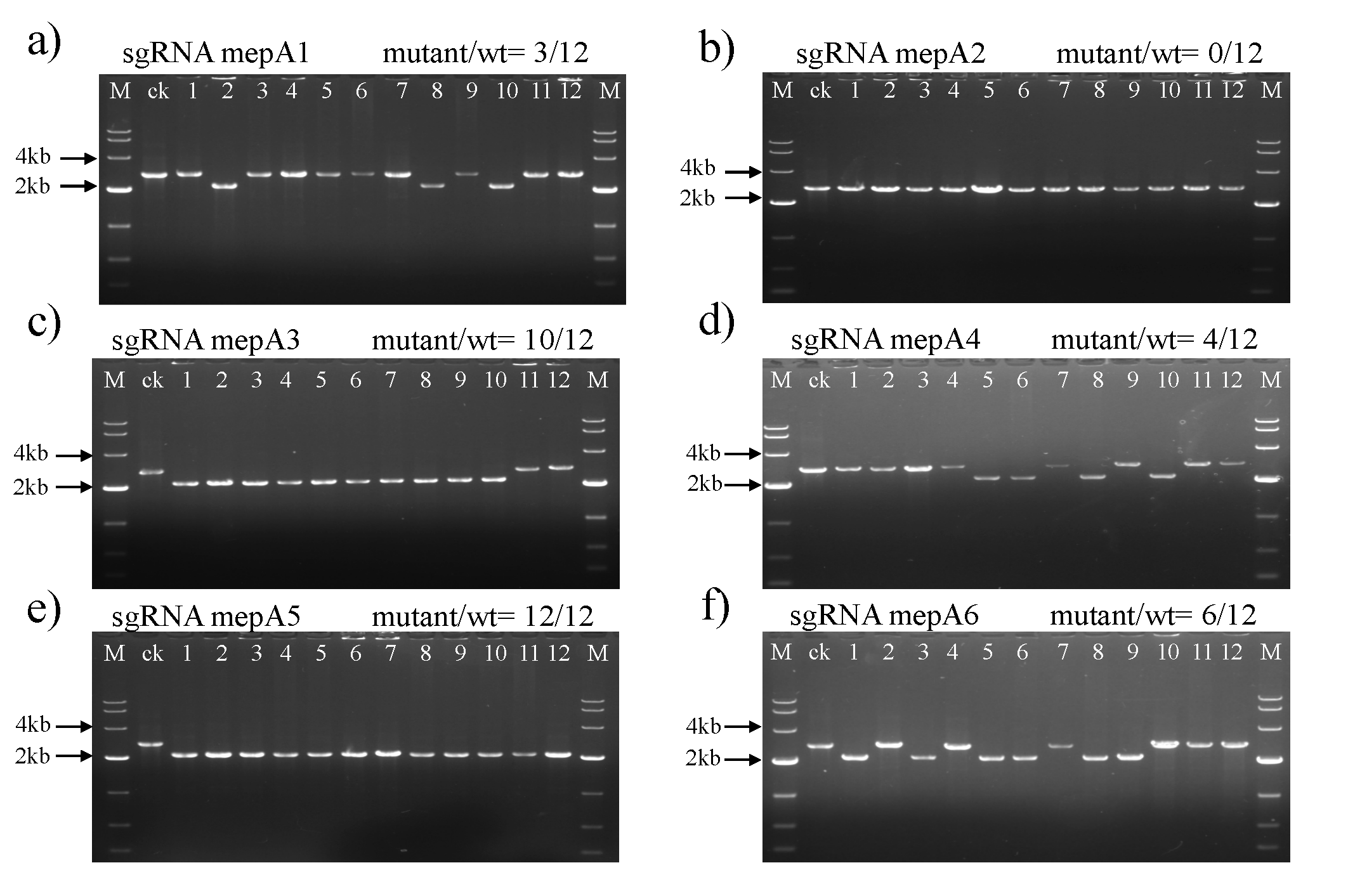


Figure S4. The map of pXMJ19-EGFP. The GFP gene was introduced into the pXMJ19 by Hind III and BamH I digestion and ligation. The *lacIq* gene was removed from PXMJ19 to make it a constitutive expression vector.

Figure S5. The map of pECXK99-gene. The gene was introduced into the pECXK99 by EcoR I and Xba I digestion and ligation. The *lacIq* gene was removed from pECXK99-gene to make it a constitutive expression vector.

Figure S6: Growth phenotypes of the *porB*, *mepA*, Ncgl0911-deleted strains and their complementation strains. WT: wild-type strain; MporB: *porB*-deleted mutant; MmepA: *mepA*-deleted mutant; M0911: Ncgl0911-deleted mutant; MmepA-mepA: *mepA*-deleted mutant complemented by *mepA* gene; MporB-porB: *porB*-deleted mutant complemented by *porB* gene; M0911-0911: Ncgl0911-deleted mutant complemented by Ncgl0911 gene. The complementation strains of *porB*, *mepA* and Ncgl0911 showed the same growth rate as the wild type and mutant strains. Date are representative of triplicate cultures.

Figure S7. The analysis of GFP expression by SDS-PAGE. CK is a negative control of the wild-type strain containing pXMJ19 without the *gfp* gene. The lane 1 is the positive control of wild-type strain with plasmid pXMJ19-EGFP.The lane 2 is the expression of GFP in *mepA* gene deleted strain. The lane3 is the expression of GFP in *mepA* gene complemented strain. The lane 4 is the expression of GFP in the *porB* gene deleted strain. The lane 5 is the expression of GFP in *porB* gene complemented strain.


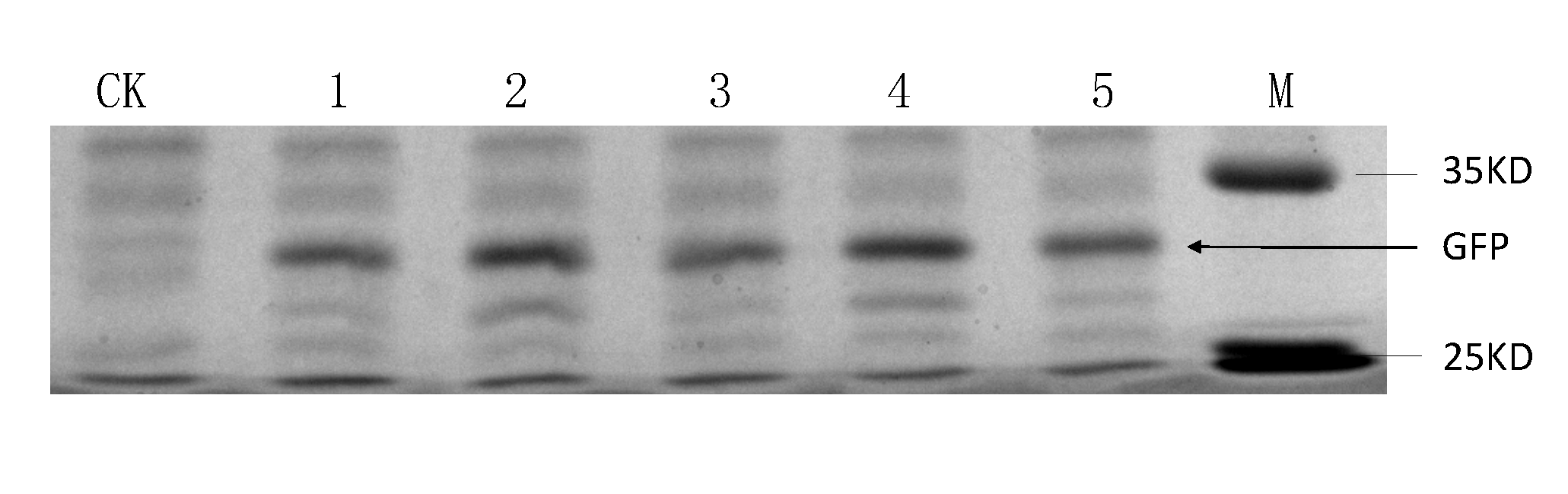


Table S1. Strains and Plasmids used in this study.

| strain | properities | source |
| --- | --- | --- |
| *C. glutamicum* ATCC 13032 | Wild type | ATCC |
| *C. glutamicum* CGMCC1.15647 | suitable to express foreign proteins | Lab stock |
| *E. coli* DH5α | Cloning strain [F– *endA1 glnV44 thi-1 recA1 relA1 gyrA96 deoR nupG purB20* φ80d*lacZ*ΔM15Δ(*lacZYA-argF*)U169 hsdR17(*rK*–*mK*+) λ–] | Lab stock |
| MporB | *C. glutamicum* ATCC 13032 with *porB* gene deletion | this study |
| MmepA | *C. glutamicum* ATCC 13032 with *mepA* gene deletion | this study |
| MclpX | *C. glutamicum* ATCC 13032 with *clpX* gene deletion | this study |
| M0911 | *C. glutamicum* ATCC 13032 with Ncgl0911 gene deletion | this study |
| MporB-porB | *C. glutamicum* ATCC 13032 *porB* gene deleted strain complemented with *porB* gene | this study |
| MmepA-mepA | *C. glutamicum* ATCC 13032 *mepA* gene deleted strain complemented with *mepA* gene | this study |
| M0911-0911 | *C. glutamicum* ATCC 13032 Ncgl0911 gene deleted strain complemented with Ncgl0911 gene | this study |
| *C. glutamicum* ATCC 13032 Cas9 | *C. glutamicum* ATCC 13032 Wild type strain containing Cas9 protein |  |

| plasmid | properities | source |
| --- | --- | --- |
| pEC-XK99E | *E. coli−C. glutamicum* Ptrc constitutive expression shuttle vector, Km | [^1^](#_ENREF_1) |
| pXMJ19 | *E. coli−C. glutamicum* Ptac constitutive expression shuttle vector, Cm | [^2^](#_ENREF_2) |
| pcas9 | plasmid carrying cas9 gene, Km | this study |
| psgRNA | plasmid carrying sgRNA scafford, Km | this study |
| PEGFP-N1 | plasmid carrying the EGFP, Amp | lab stock |
| pXMJ19-EGFP | pXMJ19 plasmid removed the *lacIq* and carrying *gfp* | lab stock |
| pFST | pEC-XK99E temperature sensitively plasmid | lab stock |
| pFSC | pXMJ19 plasmid carrying cas9 driven by the IPTG-inducide Ptac promoter , KanR | this study |
| pFST-porB | pFST plasmid carrying the *proB* sgRNA and 1000bp homologous arm of *porB* | this study |
| pFST-porB15674 | pFST plasmid carrying the *proB* sgRNA and 1000bp homologous arm of *porB* in *C. glutamicum* CGMCC1.15647 | this study |
| pFST-porBsgRNA | pFST plasmid carrying the *proB* sgRNA | this study |
| pFST-porBT | pFST plasmid carrying the *proB* homologous arm | this study |
| pFST-porb100 | pFSTplasmid carrying the *proB* sgRNA and homologous arm about 100bp of *porB* | this study |
| pFST-porB300 | pFST plasmid carrying the *proB* sgRNA and homologous arm about 300bp of *porB* | this study |
| pFST-porB600 | pFST plasmid carrying the *proB* sgRNA and homologous arm about 600bp of *porB* | this study |
| pFST-porBM | pFST plasmid carrying the *proB* sgRNA and homologous arm with point mutation site of *porB* | this study |
| pFST-porBGFP | PFST plasmid carrying the *proB* sgRNA and homologous arm with GFP insertion of *porB* | this study |
| pFST-porB300GFP | PFST plasmid carrying the *proB* sgRNA and 300bp homologous arm with GFP insertion of *porB* | this study |
| pFST-mepA | pFST plasmid carrying the *mepA* sgRNA and homologous arm of *mepA* | this study |
| pFST*-*clpX | PFST plasmid carrying the *clpX* sgRNA and homologous arm of *clpX* | this study |
| pFST-0911 | pFST plasmid carrying the 0911 sgRNA and homologous arm of 0911 | this study |
| pFST-mepA1 | pFST plasmid carrying the *mepA* sgRNA1 and homologous arm of *mepA* | this study |
| pFST-mepA2 | pFST plasmid carrying the *mepA* sgRNA2 and homologous arm of *mepA* | this study |
| pFST-mepA3 | PFST plasmid carrying the *mepA* sgRNA3 and homologous arm of *mepA* | this study |
| pFST-mepA4 | pFST plasmid carrying the *mepA* sgRNA4 and homologous arm of *mepA* | this study |
| pFST-mepA5 | pFST plasmid carrying the *mepA* sgRNA5 and homologous arm of *mepA* | this study |
| pFST-mepA6 | pFST plasmid carrying the *mepA* sgRNA6 and homologous arm of *mepA* | this study |
| pECXK99-porB | pECSK99 plasmid removed the *lacIq* and carrying *porB* | this study |
| pEXCK99-mepA | pECXK99 plasmid removed the *lacIq* and carrying *mepA* | this study |
| pEXCK99-0911 | pECXK99 plasmid removed the *lacIq* and carrying Ncgl0911 | this study |

Table S2. Primers used in this study.

|  | name | sequnence | description |
| --- | --- | --- | --- |
| pFCS plasmid construction | cas9F | CGCAAGCTTAAAGGAGGACAACTAATGGATAAAAAGTATTC | amplification of the cas9 gene with SD sequnence |
|  | cas9R | TATGAATTCTTAGTCGCCACCCAGCTGGGAGAGA | amplification of the cas9 gene with SDsequnence |
| pFTS plasmid construction | sgRNAporBF | TGGAATTCGGAGGATAGGTTTGCGAAGTGTTTTAGAGCTAGAA | amplification the *porB* sgRNA |
|  | sgRNAmepAF | TGGAATTCGGCACCT TCACCTCAGGATTGTTTTAGAGCTAGAA | amplification the *mepA* sgRNA |
|  | sgRNAclpXF | TGGAATTCGGCTGAAATCTCCGACGGCTGTTTTAGAGCTAGAA | amplification the *clpX* sgRNA |
|  | sgRNA0911F | TGGAATTCGGTAACTGGGCTGGCCAAAAGTTTTAGAGCTAGAA | amplification the 0911 sgRNA |
|  | sgRNAmepA1F | TGGAATTCGGTGGCGGTAGCGGTTGCGGGTTTTAGAGCTAGAA | amplification the *mepA*1 sgRNA |
|  | sgRNAmepA2F | TGGAATTCGGATGCTGGTGCCATGGTGGGTTTTAGAGCTAGAA | amplification the *porB* sgRNA |
|  | sgRNAmepA3F | TGGAATTCGGAGGAAAGGCCTGCGTAGTGTTTTAGAGCTAGAA | amplification the *porB* sgRNA |
|  | sgRNAmepA4F | TGGAATTCGGCGGCTGGTGCGACGGCGGGTTTTAGAGCTAGAA | amplification the *porB* sgRNA |
|  | sgRNAmepA5F | TGGAATTCGGCCAGGAAATCGCAGGAATGTTTTAGAGCTAGAA | amplification the *porB* sgRNA |
|  | sgRNAmepA6F | TGGAATTCGGCAGCCAAGGATTCTCCACGTTTTAGAGCTAGAA | amplification the *porB* sgRNA |
|  | sgRNAR | ACTCTAGAATTTAATCTGTATCAGGCTG | amplification the sgRNA |
| *porB* gene deletion in ATCC13032 | porBLF | CTGATGGCGCAGGGGATCAACAAGTCACGATCGTTGGAAC | amplification the 1kb *porB* upstream for gene deletion |
|  | porBLR | GAACCTTTTAGATTTTTAGGGCTCCTTTTAAGG | amplification the 1kb *porB* upstream for gene deletion |
|  | porBRF | CCTAAAATCTAAAAGGTTCGGGGGTAAC | amplification the 1kb *porB* downstream for gene deletion |
|  | porBRR | CTCATCCTGTCTCTTGATCAATCGATCCCGATTTTC | amplification the 1kb *porB* downstream for gene deletion |
|  | porB600LF | CTGATGGCGCAGGGGATCAAATCAGATCTCCTGCCAAGGTATTCCAC | amplification the 600bp *porB* upstream for gene deletion |
|  | porB600RR | CTCATCCTGTCTCTTGATCAAGCAGATCTGTTTCGTAGCCAAGATGC | amplification the 600bp *porB* upstream for gene deletion |
|  | porB300LF | CTGATGGCGCAGGGGATCAAATCAGATCTTGAGGGTCGATGTTACCG | amplification the 300bp *porB* downstream for gene deletion |
|  | porB300RR | CTCATCCTGTCTCTTGATCAATCAGATCTGCTCTGAGCGAGTGGAGTT | amplification the 300bp *porB* downstream for gene deletion |
| *porB* gene deletion in CGMCC1.15647 | porBbzhLF | CTGATGGCGCAGGGGATCAAGGAGTGGACAAGTCACGAT | amplification the 1kb *porB* upstream for gene deletion |
|  | porBbzhLR | TTGGAGGACATGCCACTGTGATGCCTGCGGTTGCT | amplification the 1kb *porB* upstream for gene deletion |
|  | porBbzhRF | CACAGTGGCATGTCCTCCAACTTCTCTTCCTAAAA | amplification the 1kb *porB* downstream for gene deletion |
|  | porBbzhRR | CTCATCCTGTCTCTTGATCAGGGTGAACCTGTTTCTATC | amplification the 1kb *porB* downstream for gene deletion |
| *porB* gene point mutation and gfp gene insertion | porBMLF | CTGATGGCGCAGGGGATCAAGTCTTTCTAACCAAAAGACC | amplification the *proB* fragment with mutation site |
|  | porBMLR | GGTTTAGTATCTCGGATG | amplification the *proB* fragment with mutation site |
|  | porBMRF | ATCCGAGATACTAAACCTATC | amplification the *proB* fragment with mutation site |
|  | porBMRR | CTCATCCTGTCTCTTGATCAGAACCTTTTAGGAAGAGAAG | amplification the *proB* fragment with mutation site |
|  | porBILF | CTGATGGCGCAGGGGATCAACAAGTCACGATCGTTGGAAC | amplification the 1kb *proB* upstream for gene insertion |
|  | porBILR | GGCATGATAGGATTTTTAGGGCTCCTTTTAAGG | amplification the *proB* upstream for gene insertion |
|  | gfpF | CCTAAAAATCCTATCATGCCATACCGCG | amplification the gfp gene for gene insertion |
|  | gfpR | CCCGAACCTTTTACTTGTACAGCTCGTC | amplification the gfp gene for gene insertion |
|  | porBIRF | GTACAAGTAAAAGGTTCGGGGGTAACCC | amplification the *proB* downstream for gene insertion |
|  | porBIRR | CTCATCCTGTCTCTTGATCATCTATCGATCCCGATTTTCCATTTG | amplification the 1kb *proB* downstream for gene insertion |
|  | porBILF300 | CTGATGGCGCAGGGGATCAAATCAGATCTTGAGGGTCGATGTTACCG | amplification the 300bp *proB* upstream for gene insertion |
|  | porBIRR300 | CTCATCCTGTCTCTTGATCAATCAGATCTGCTCTGAGCGAGTGGAGTT | amplification the 300bp *proB* downstream for gene insertion |
| *mepA* gene deletion | mepALF | CTGATGGCGCAGGGGATCAATTTGCGGCGGTGCCCTCC | amplification the 300bp *mepA* upstream for gene deletion |
|  | mepALR | CTAGCGCTTAGAGAATCCTTTTTATGGAGGTTAAAATGGTCAGATTGGC | amplification the 300bp *mepA* upstream for gene deletion |
|  | mepARF | AAGGATTCTCTAAGCGCTAGCCGTTCGTG | amplification the 300bp *mepA* downstream for gene deletion |
|  | mepARR | CTCATCCTGTCTCTTGATCAGGCTCGCTCATAGAGTGTTATTC | amplification the 300bp *mepA* downstream for gene deletion |
| *clpX* gene deletion | clpXLF | CTGATGGCGCAGGGGATCAAGGAGGTGGCGGTGGTGCG | amplification the 300bp *clpX* upstream for gene deletion |
|  | clpXLR | TGCCAACTACTAGACTGTGAGACCCGCTTCCAC | amplification the 300bp *mepA* upstream for gene deletion |
|  | clpXRF | TCACAGTCTAGTAGTTGGCAGGAGTTATCACCG | amplification the 300bp *clpX* downstream for gene deletion |
|  | clpXRR | CTCATCCTGTCTCTTGATCATCCTGTCGCCGTAGTGGC | amplification the 300bp *clpX* downstream for gene deletion |
| Ncgl0911 gene deletion | 0911LF | CTGATGGCGCAGGGGATCAAGGATCAATCCGCTTCGTATTC | amplification the 300bp 0911 upstream for gene deletion |
|  | 0911LR | GAAATCATTACGTGTCTCAATGTTAGCTG | amplification the 300bp 0911 upstream for gene deletion |
|  | 0911RF | TTGAGACACGTAAATGATTTCCATTTCCATCGCCGACG | amplification the 300bp 0911 downstream for gene deletion |
|  | 0911RR | CTCATCCTGTCTCTTGATCACTGCTGCAAGCGCGCGTT | amplification the 300bp 0911 downstream for gene deletion |
| plasmid insertion site PCR and sequencing | pecBglF | ACGCCCTGTGAGTTCCC | PCR verification of the fragment insertion |
|  | pecBglR | CAGCCGATTGTCTGTTGTG | PCR verification of the fragment insertion |
|  | p19F | CAGTGAGACGGGCAACA | PCR verification and sequencing of the cas9 |
|  | p19R | AGGGAATAAGGGCGACA | PCR and quenching verification of the cas9 |
|  | pECF | ATTCGTGTCGCTCAAGGC | sequencing verification of the sgRNA |
|  | porBseq | ATCAGATCTCCTGCC | sequencing verification of the *porB* gene deletion |
|  | PorBseq15674 | GCAATTAGTAGAGCATTC | sequencing verification of the *porB* gene deletion in C. glutamicum CGMCC1.15647 |
|  | mepAseq | TTTGCGGCGGTGCCCTCC | sequencing verification of the *mepA* gene deletion |
|  | clpXseq | GGAGGTGGCGGTGGTGCG | quenching verification of the *clpX* gene deletion |
|  | Ncgl0911seq | GGATCAATCCGCTTCGTATTC | quenching verification of the Ncgl0911 gene deletion |
| Genetic complementation | mepAF | GCTGAATTCATGCTAAACAT | amplification the *porB* gene for complementation |
|  | mepAR | CACTCTAGATAGCGCTTAAA | amplification the *porB* gene for complementation |
|  | porBF | GAGGAATTCATGAAGCTTTC | amplification the *mepA* gene for complementation |
|  | porBR | CGCTCTAGATTAGGAAGAGA | amplification the *mepA* gene for complementation |
|  | 0911F | GAGGAATTCATGAATAAAGA | amplification the Ncgl0911 gene for complementation |
|  | 0911R | CGCTCTAGATTAAGCTTCCT | amplification the Ncgl0911 gene for complementation |

Table S3. Off target analysis of edited strains.

|  | *C. glutamicum*  ATCC 13032 Cas9 | *C. glutamicum*  ATCC 13032 MmepA | *C. glutamicum*  ATCC 13032 MporB |
| --- | --- | --- | --- |
| Genome size(bp) | 3,268,055 | 3,273,516 | 3,273,674 |
| Hiseq date(Mb) | 350 | 344 | 348 |
| CDS SNP | 0 | 0 | 0 |
| Intergenic SNP | 0 | 0 | 0 |
| CDS Indel | 0 | 0 | 0 |
| Intergenic Indel | 0 | 0 | 1 |

Summary of variant type found in *C. glutamicum* ATCC 13032 wild type containing Cas9 protein, *C. glutamicum* ATCC 13032 *mepA*-deleted strain and *C. glutamicum* ATCC 13032 *porB*-deleted strain. Genome size is the genome size of each sample; Hiseq date is the date size of High-throughput sequencing for each sample; CDS SNP is the number of SNP occurred in CDS regions; Intergenic SNP is the number of SNP generated in intergenic region; CDS Indel and Intergenic Indel are the number of Indel generated in CDS regions and Intergenic region, respectively.

References

(1) Kirchner, O.; Tauch, A. (2003) Tools for genetic engineering in the amino acid-producing bacterium *Corynebacterium glutamicum*. *J. Biotechnol.***,** *104* (1), 287-299.

(2) Jakoby, M.; Ngouoto-Nkili, C.-E.; Burkovski, A. (1999) Construction and application of new *Corynebacterium glutamicum* vectors. *Biotechnol. Tech.***,** *13* (6), 437-441.
